# MiRNAs expression profiling of rat ovaries displaying PCOS with insulin resistance

**DOI:** 10.1007/s00404-020-05730-z

**Published:** 2020-08-05

**Authors:** Chunren Zhang, Chuyi Yu, Zengxian Lin, Haixia Pan, Kunyin Li, Hongxia Ma

**Affiliations:** 1 The Third Affiliated Hospital of Guangzhou University of Chinese Medicine, Guangzhou, 510240 Guangdong China; 2 Department of Traditional Chinese Medicine, First Affiliated Hospital of Guangzhou Medical University, No.151 Yanjiang Road, Yuexiu District, Guangzhou, 510120 Guangdong China; 3 Artemisinin Research Center, Guangzhou University of Chinese Medicine, Guangzhou, 510405 China

**Keywords:** MicroRNAs, High-throughput sequencing, Polycystic ovary syndrome, Insulin resistance

## Abstract

**Purpose:**

The present study established microRNA (miRNA) expression profiles for rat ovaries displaying polycystic ovary syndrome (PCOS) with insulin resistance and explored the underlying biological functions of differentially expressed miRNAs.

**Methods:**

A PCOS with insulin resistance rat model was created by administering letrozole and a high-fat diet. Total RNA was extracted from the ovaries of PCOS with insulin resistance rats and normal rats. Three ovaries from each group were used to identify differentially expressed miRNAs by deep sequencing. A hierarchical clustering heatmap and volcano plot were used to display the pattern of differentially expressed miRNAs. Gene ontology (GO) analysis and Kyoto Encyclopedia of Genes and Genomes (KEGG) pathway analysis were conducted to explore the potential target genes of the differentially expressed miRNAs and identify their putative biological function. Nine of the differentially expressed miRNAs were selected for validation by Real-time Quantitative PCR (qRT-PCR).

**Results:**

A total of 58 differentially expressed miRNAs were identified in the rat ovaries exhibiting PCOS with insulin resistance compared with control ovaries, including 23 miRNAs that were upregulated and 35 miRNAs that were downregulated. GO and KEGG pathway analyses revealed that the predicted target genes were related to metabolic processes, cellular processes, and metabolic pathways. Furthermore, qRT-PCR confirmed that miR-3585-5p and miR-30-5p were significantly upregulated and miR-146-5p was downregulated in the ovaries of PCOS with insulin resistance rats compared with the controls.

**Conclusion:**

These results indicate that differentially expressed miRNAs in rat ovaries may be involved in the pathophysiology of insulin resistance in PCOS. Our study may be beneficial in establishing miRNAs as novel diagnostic and therapeutic biomarkers for insulin resistance in PCOS.

## Introduction

Polycystic ovary syndrome (PCOS), which affects 6–20% of women during reproductive age, is one of the most common endocrine and metabolic disorders [1–3]. According to the Rotterdam criteria, PCOS may be diagnosed with at least two of the three following characteristics: anovulation, clinical and/or biochemical signs of hyperandrogenism and polycystic ovaries [4, 5]. PCOS is also associated with infertility, insulin resistance, obesity, glucose tolerance, dyslipidemia, type 2 diabetes and increased risk for cardiovascular disease [6]. Insulin resistance and hyperandrogenism are not only the two main characteristic features of PCOS, but also important pathogenic factors for PCOS [7]. Furthermore, there are approximately 40–70% of PCOS patients with insulin resistance. Insulin may promote luteinizing hormone release, increase androgen levels by ovarian theca cells, and inhibit the production of sex hormone binding protein by the liver, resulting in elevated levels of circulating free testosterone. Therefore, insulin synergizing with androgen could result in deteriorating anovulation, hyperandrogenism, hyperinsulinemia and infertility in PCOS patients. However, whether insulin resistance is the cause or the result of PCOS remains to be determined. Genetic studies and familial clustering of phenotypic features have indicated that PCOS is heritable [8–11]. Genome-wide association studies have found over 20 PCOS susceptibility genes; however, they account for < 10% of all PCOS cases [12–16]. Therefore, other mechanisms must play a role in the development of PCOS as genetic factors are responsible for only a small portion of the cases. One potential mechanism is an epigenetic process which could yield the same phenotypic characteristics as heritable genetics [17, 18].

MicroRNAs (miRNAs) are small, single-stranded noncoding RNAs consisting of 18–24 nucleotides in length. They regulate post-transcriptional gene expression by binding to the 3′ untranslated region of target RNAs [19]. MiRNAs are involved in many physiological processes including cell growth, differentiation, proliferation and metabolism [20]. Furthermore, several studies have shown that dysfunctional miRNAs are also associated with the pathological mechanism of PCOS [21]. Sang et al. identified highly expressed miRNAs in human follicular fluids [22]. In addition, Chen et al. found that miR-93 is upregulated in the adipose tissue of PCOS patients and women with insulin resistance [23]. In our previous study, we found differentially expressed circular RNAs (circRNAs) in the granulosa cells of reproductive-aged PCOS women [24]. However, there is no literature regarding miRNA expression profiling in the ovaries of PCOS patients with insulin resistance. In the present study, we identified differentially expressed miRNAs from PCOS ovaries in an insulin-resistant rat model and determined their putative function.

## Materials and methods

### Animals and treatment

All animal experiments were carried out based on the Guidelines for the Care and Use of Experimental Animals and approved by the Animal Ethics Committee of the Guangzhou University of Chinese Medicine (20180715002). Three-week-old female Sprague Dawley rats were purchased from the Animal Centre of Guangdong Province (Guangdong, China). Animals were fed adaptively for 1 week under conditions of 55–65% humidity, 21–22 °C temperature, and a 12 h light/12 h dark cycle. Twenty rats were randomly divided into two groups. Ten rats in the control group (CON) were fed a normal diet (Research Diet, D12450, 10% fat), while 10 rats in the treatment group were fed a high-fat diet (Research Diet, D12492, 60% fat) for 8 weeks (LEHF group). In addition, the con rats were gavaged with 1% carboxymethylcellulose (CMC) solution once daily, and LEHF rats were gavaged with 1 mg/kg letrozole in 1% CMC once daily for 21 days [25]. All rats were weighed each week.

### Estrus cycle

The estrus cycle was examined daily. Depending on the results of microscopic analysis of a vaginal smear, the animals were flushed with physiological saline from the 12th day to the end of the experiment.

### Blood and tissue sampling

All rats were anesthetized with isoflurane after overnight fasting. Blood samples were collected from the heart and the resulting serum samples were stored at – 80 °C. Serum levels of follicle stimulating hormone (FSH), luteinizing hormone (LH), estradiol (E2), testosterone (T), progesterone (P), fasting blood glucose (FBG) and fasting insulin (FINS) were measured using ELISA kits (Cloud-Clone Corp., Wuhan, China) according to the manufacturer’s protocol. The homeostasis model assessment for insulin resistance (HOMA-IR) was calculated as previously described using the following formula: HOMA-IR = (FBG × FINS) / 22.5 [26].

Ovary samples were quickly collected from the animals, and one ovary from each rat was fixed in 4% paraformaldehyde. The ovary tissue samples were then dehydrated, embedded in paraffin, and sectioned into 4 µm sections. Each section was stained with hematoxylin and eosin (H&E). The other rat ovary was stored at – 80 °C and used to establish miRNA expression profiles.

### RNA-sequencing (RNA-seq) and bioinformatics analysis

RNA isolation and RNA sequencing were performed as previously described [24]. Briefly, total RNA was extracted from ovaries using TRIzol reagent according to the manufacturer’s instructions. The concentration of RNA was measured with a NanoDrop 2000 spectrophotometer. An RNA library was generated from total RNA using the NEB Next Ultra Directional RNA Library Prep Kit for Illumina (NEB, MA, USA). The RNA library quality and quantity were assessed with an Agilent 2100 Bioanalyzer and an ABI Step One Plus Real-Time PCR System, respectively. The RNA library was run on a HiSeq 2000 platform (Illumina, CA, USA) for sequence analysis. The clean reads were filtered from raw reads by FastQC and used for further bioinformatics analysis. The differentially expressed miRNAs were selected based on log_2_ (LEHF/CON) > 1 and *P* value < 0.05.

Hierarchical clustering heatmaps and volcano plots generated by R package version 1.0.8 software (https://cran.r-project.org/web/packages/pheatmap/) were used to display the differentially expressed miRNA patterns between the two groups. Gene ontology (GO) analysis was performed to explore the molecular function, cellular components and biological processes of the differentially expressed miRNAs (https://www.geneontology.org). The biological pathways of the differentially expressed miRNAs were further analyzed using the Kyoto Encyclopedia of Genes and Genomes (KEGG; https://www.genome.jp/kegg/) database.

### Real-time quantitative PCR (qRT-PCR)

To verify the results of RNA sequencing, we measured relative miRNA expressions by qRT-PCR analysis using SYBR Premix Ex Taq II (Takara, China). The *U6* gene was used as an internal control. The relative expression of miRNAs was calculated using the comparative Ct (2^−ΔΔCT^) method [27]. The sequences of primers used in this study are shown in Table 1.

**Table 1:** The primer sequences in this study

Gene name	Primer sequences (5′-3′)
rno-miR-3585-5p-RT	GTCGTATCCAGTGCAGGGTCCGAGGTATTCGCACTGGATACGACATGAAA
rno-miR-3585-5p-F	AACACGCTTCACAAGAAGGTG
rno-miR-200a-3p-RT	GTCGTATCCAGTGCAGGGTCCGAGGTATTCGCACTGGATACGACACATCG
rno-miR-200a-3p-F	AACACGCTAACACTGTCTGGT
rno-miR-30a-5p-RT	GTCGTATCCAGTGCAGGGTCCGAGGTATTCGCACTGGATACGACCTTCCA
rno-miR-30a-5p-F	AACACGCTGTAAACATCCTCG
rno-miR-134-5p-RT	GTCGTATCCAGTGCAGGGTCCGAGGTATTCGCACTGGATACGACCCCCTC
rno-miR-134-5p-F	AACAGTGTGTGACTGGTTGAC
rno-miR-132-3p-RT	GTCGTATCCAGTGCAGGGTCCGAGGTATTCGCACTGGATACGACCGACCA
rno-miR-132-3p-F	AACACGCTAACAGTCTACAGC
rno-miR-146b-5p-RT	GTCGTATCCAGTGCAGGGTCCGAGGTATTCGCACTGGATACGACACAGCC
rno-miR-146b-5p-F	AACACGCTGAGAACTGAATTCC
rno-miR-21-5p-RT	GTCGTATCCAGTGCAGGGTCCGAGGTATTCGCACTGGATACGACTCAACA
rno-miR-21-5p-F	AAGCGACCTAGCTTATCAGACT
rno-miR-124-3p-RT	GTCGTATCCAGTGCAGGGTCCGAGGTATTCGCACTGGATACGACGGCATT
rno-miR-124-3p-F	TAAGGCACGCGGTGAA
rno-miR-122-5p-RT	GTCGTATCCAGTGCAGGGTCCGAGGTATTCGCACTGGATACGACCAAACA
rno-miR-122-5p-F	AACCGGTGGAGTGTGACAAT
rno-miR-375-3p-RT	GTCGTATCCAGTGCAGGGTCCGAGGTATTCGCACTGGATACGACTCACGC
rno-miR-375-3p-F	AACACGCTTTGTTCGTTCGG

### Statistical analysis

All values are presented as the mean ± SD. Comparisons between experimental and control groups were conducted by a Student’s *t* test. Data analyses were performed using SPSS 19.0 software (SPSS Inc., IL, USA). *P* value < 0.05 was considered statistically significant.

## Results

### Animal model of PCOS with insulin resistance

**Fig.1:**
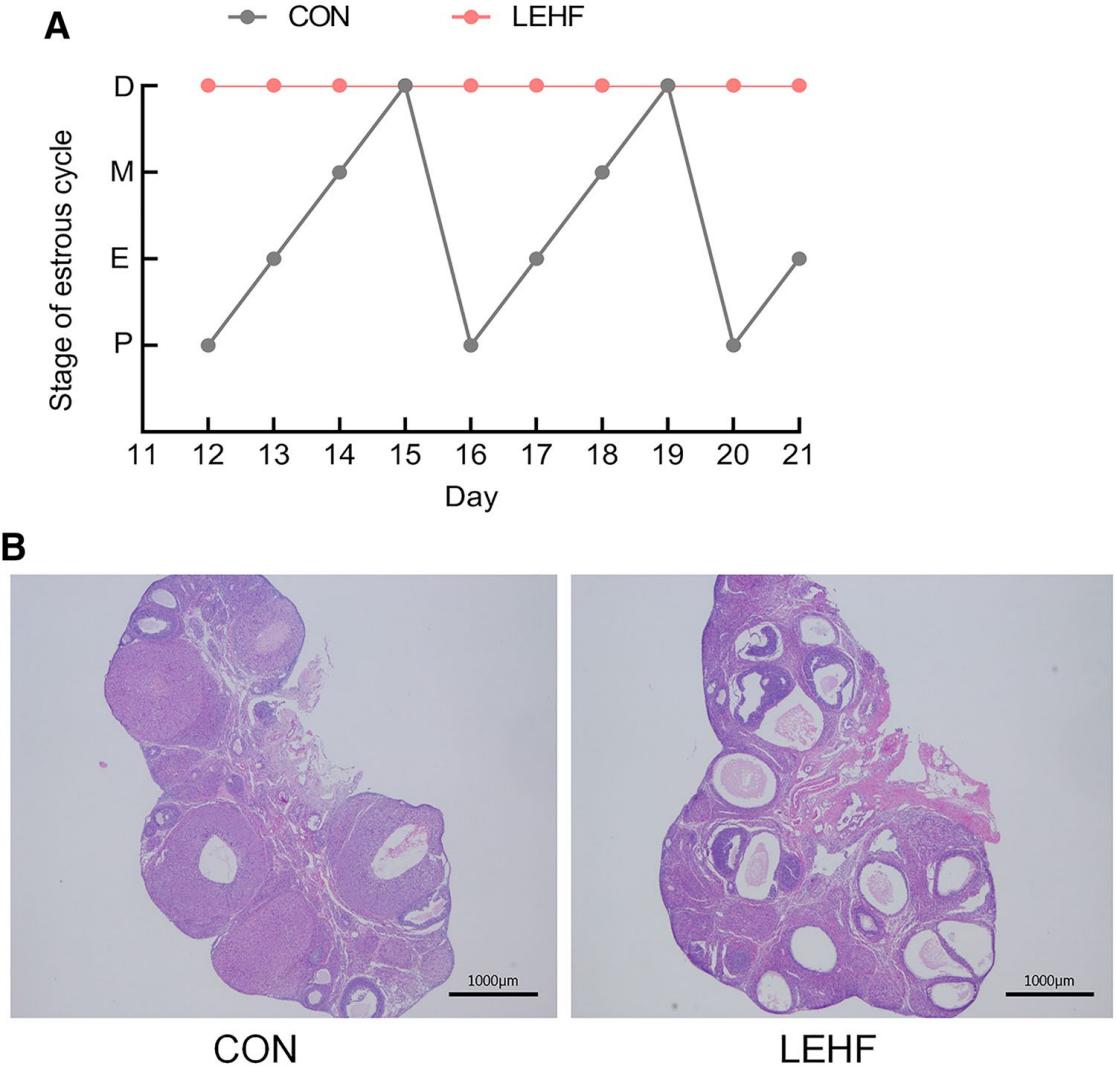
Change in estrous cycles and morphology of ovaries of rats from the LEHF-induced PCOS with insulin resistance and the groups. **a** Changes in estrous cycle between the LEHF and CON groups. **b** Morphological changes in the rat ovarian tissues as detected by H&E. *LEHF* letrozole and high-fat diet group, *D* diestrus, *P* proestrus, *E* estrus, *M* metestrus

**Fig.2:**
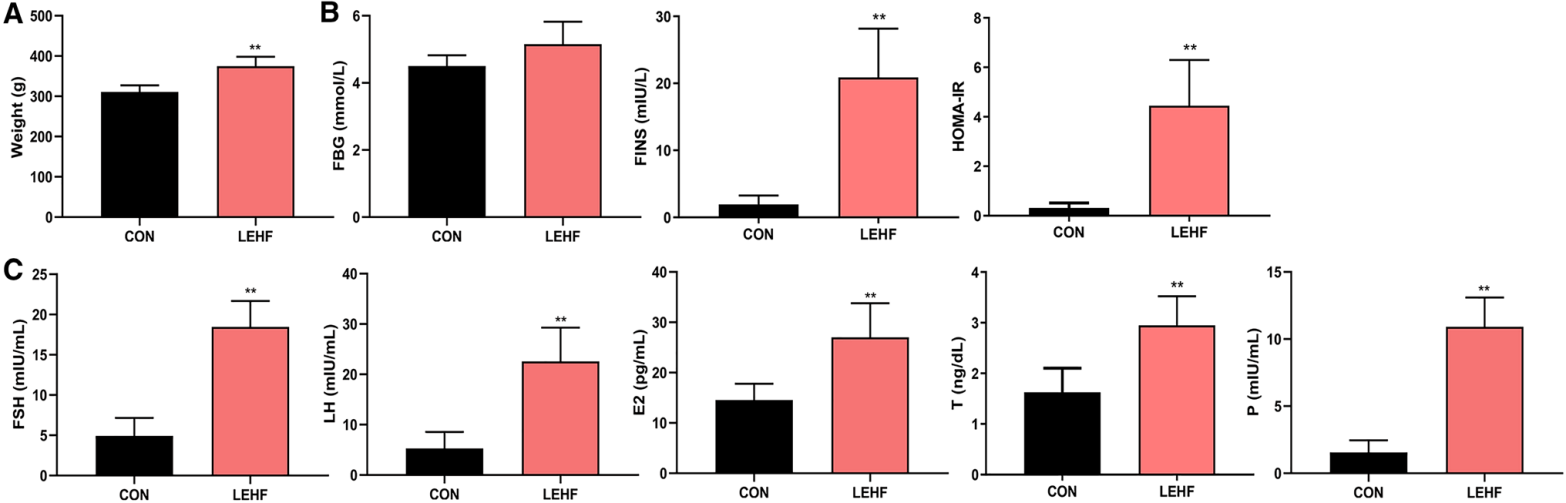
Changes in blood sugar and hormone levels between the LEHF and CON groups. **a** Comparison of weight between the two groups. **b** The values of FBG, FINS and HOMA-IR were determined for the two groups. **c** The LH, FSH, E2, T and P levels were determined for the two groups. *P < 0.05; **P < 0.01. *FBG* fasting blood glucose, *FINS* fasting insulin, *HOMA-IR* homeostasis model assessment of insulin resistance, *FSH* follicle stimulating hormone, *LH* luteinizing hormone, *E2* estradiol, *T* testosterone, *P* progesterone

We used letrozole and a high-fat diet to establish a rat model with a PCOS and insulin-resistance phenotype. The CON rats exhibited a normal estrus cycle, whereas the LEHF rats had a disrupted estrous cycle (Fig. 1a). H&E staining results showed that the ovary in the CON group had follicles and corpora lutea at various stages of development, a normal theca and granulosa cell layer and no ovarian cysts. In the LEHF group, the ovary exhibited atretic antral follicles, more large cystic follicles with a thinner granulosa cell layer and fewer corpora lutea (Fig. 1b). Compared with the CON group, the body weight, FINS and HOMA-IR were significantly increased in rats from the LEHF group (Fig. 2a, b). Moreover, rats in the LEHF group exhibited a dramatic increase in the LH, FSH, E2, T and P levels (Fig. 2c). Taken together, these results indicated that we successfully established a rat PCOS model with insulin resistance.

### Differentially expressed miRNA profiles in the ovary of the LEHF and CON groups

**Fig.3:**
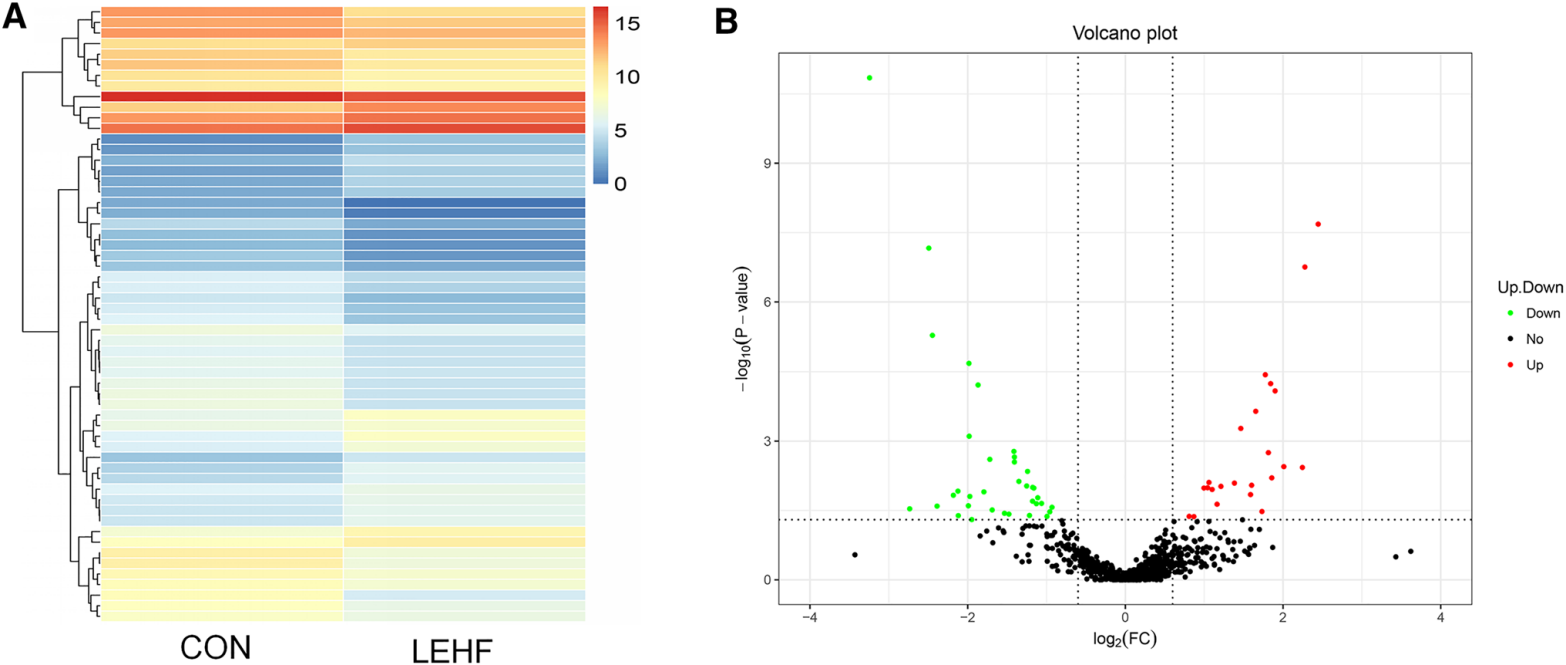
Differentially expressed miRNAs were identified by deep sequencing. A hierarchical clustering heatmap (**a**) and a volcano plot (**b**) were used to display the differentially expressed miRNA patterns between the two groups

We conducted high-throughput sequencing to establish miRNA expression profiles for three ovary samples from the LEHF and CON groups. A total of 767 miRNAs were detected in the ovary samples. Among these, 58 differentially expressed miRNAs were found between the two groups (fold change ≥ 2 and *P* value < 0.05). Among the differentially expressed miRNAs, 23 were upregulated, while 35 were significantly downregulated in the LEHF group. The list of the top 10 differentially expressed miRNAs identified by deep sequencing is shown in Table 2. A hierarchical clustering heatmap (Fig. 3a) indicated that the expression of miRNAs in the LEHF group was significantly different from that of the CON group. A volcano plot (Fig. 3b) also displayed the pattern of upregulated and downregulated miRNAs.

**Table 2:** The list of the top ten differentially expressed miRNA identified by the deep sequencing

miRNA_ID	CON	LEHF	Log_2_ fold change (LEHF/CON)	Up-down-regulation (LEHF/CON)	*P* value	FDR
rno-miR-509-3p	46.38242401	252.6640226	2.445570126	Up	2.07E-08	7.94E-06
rno-miR-547-3p	3018.488892	14629.32712	2.276965017	Up	1.75E-07	3.36E-05
rno-miR-547-5p	74.34761696	254.435207	1.774939909	Up	3.67E-05	0.004016012
rno-miR-201-5p	43.16871548	154.8170877	1.842506645	Up	5.72E-05	0.005207537
rno-miR-201-3p	15.06913716	56.21809675	1.8994378	Up	8.21E-05	0.006294168
rno-miR-3585-5p	159.4036017	501.4001728	1.653278266	Up	0.000226462	0.015790557
rno-miR-652-3p	303.3559719	837.049714	1.464301597	Up	0.000532474	0.034033945
rno-miR-200c-3p	4.111515619	14.45494958	1.813821362	Up	0.001779502	0.090991889
rno-miR-224-3p	3.472553993	13.9860925	2.00992392	Up	0.003568794	0.142581276
rno-miR-3596b	1.989015013	9.43033931	2.245255516	Up	0.003717895	0.142581276
rno-miR-132-5p	369.5014638	38.99406342	− 3.244253674	Down	1.42E-11	1.09E-08
rno-miR-132-3p	702.1346736	124.6887802	− 2.49341612	Down	6.83E-08	1.75E-05
rno-miR-146b-5p	10354.12271	1898.054539	− 2.447611968	Down	5.26E-06	0.000806138
rno-miR-212-5p	109.3275286	27.6345511	− 1.984111634	Down	2.08E-05	0.002654303
rno-miR-292-5p	308.9159765	84.59455768	− 1.868577729	Down	6.11E-05	0.005207537
rno-miR-291a-3p	98.98330743	25.07270548	− 1.98106757	Down	0.000785138	0.046323121
rno-miR-183-5p	1512.932233	567.7884137	− 1.413922053	Down	0.001674859	0.090991889
rno-miR-3553	2817.78701	1062.511993	− 1.40708344	Down	0.002209313	0.105908965
rno-miR-1298	83.26407405	25.28723408	− 1.719284907	Down	0.002478859	0.111840282
rno-miR-219a-2-3p	65.61026414	24.74143541	− 1.406992327	Down	0.002836267	0.120856493

### Functional analysis of differentially expressed miRNAs

**Fig.4:**
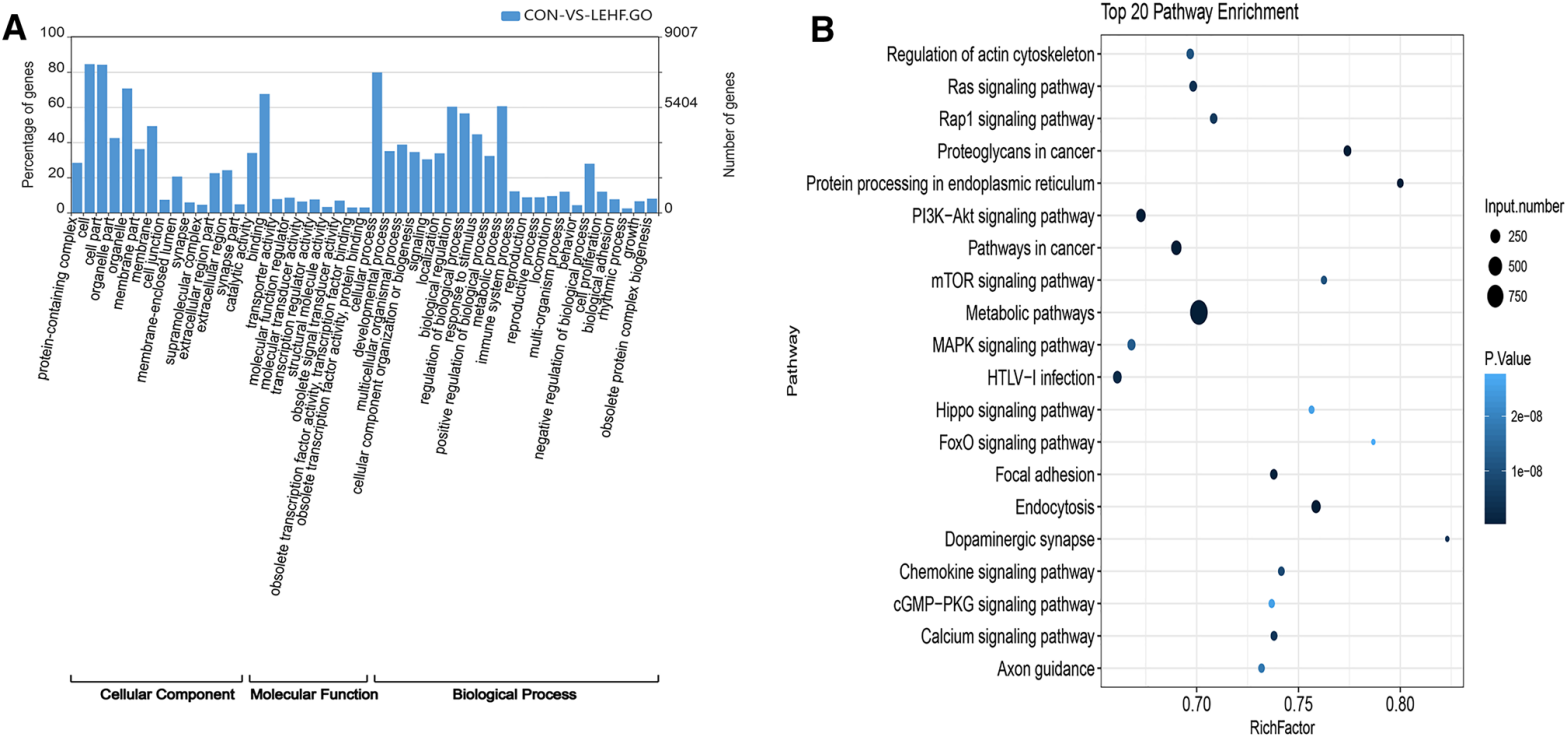
Gene ontology (GO) and KEGG pathway analysis for predicted targets of the differentially expressed miRNAs

Ovary dysfunction and abnormal follicular development are two characteristics of PCOS that are aggravated by insulin resistance. To gain insight into the underlying mechanism of PCOS with insulin resistance, GO analysis was conducted to identify the biological processes, cellular components and molecular functions of the differentially expressed miRNAs (Fig. 4a). We discovered that the most enriched GO term for the cellular component was cell apart (GO:0044464), the most enriched term for the molecular function was cellular process (GO:0009987) and the most enriched term for the biological process was metabolic process (GO:0008152). Moreover, the metabolic pathway (path: rno01100) was the most enriched pathway in the KEGG database (Fig. 4b). The biological functions and pathways for the differentially expressed miRNAs are closely related to the development of insulin resistance in PCOS.

### Validation of candidate miRNAs by qRT-PCR

**Fig.5:**
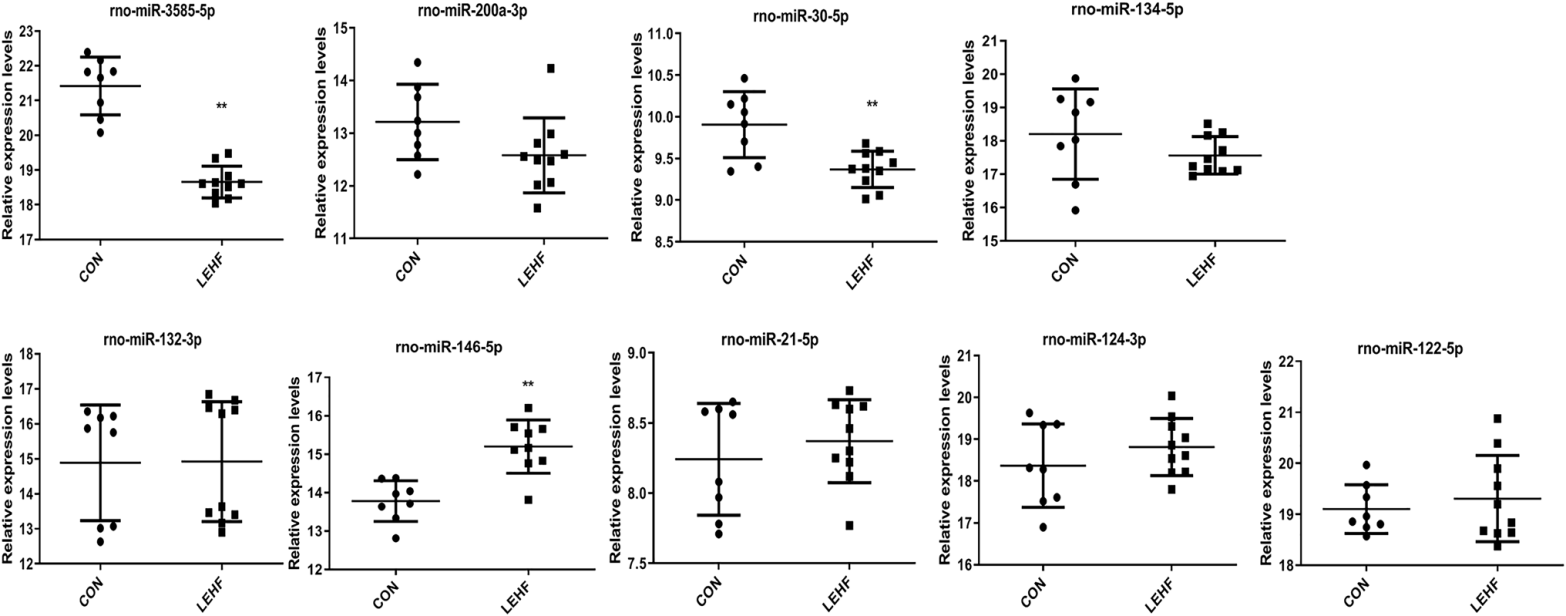
Validation of the miRNAs in the rat ovaries between the two groups by qRT-PCR. The expression of four upregulated miRNAs (miR-3585-5p, miR-200-3p, miR-30-5p and miR-134-3p) and five downregulated miRNAs (miR-132-3p, miR-146-5p, miR-21-5p, miR-124-3p and miR-122-5p) identified by deep sequencing were determined. *P < 0.05; **P < 0.01

According to the literature and the fold-change of the deep sequencing results, four upregulated miRNAs (miR-3585-5p, miR-200-3p, miR-30-5p and miR-134-3p) and five downregulated miRNAs (miR-132-3p, miR-146-5p, miR-21-5p, miR-124-3p and miR-122-5p) were selected for validation using qRT-PCR [28, 29]. As shown in Fig. 5, qRT-PCR confirmed that compared with the CON group, miR-3585-5p and miR-30-5p were significantly upregulated, while miR-146-5p was downregulated in the rat ovaries of the LEHF group. These results were consistent with the sequencing results. However, two upregulated miRNAs (miR-200-3p and miR-134-3p) and four downregulated miRNAs (miR-132-3p, miR-21-5p, miR-124-3p and miR-122-5p) were not significantly expressed or inconsistently expressed as compared with the sequencing results.

## Discussion

PCOS is a complex heterogeneous disorder characterized by hyperandrogenism and insulin resistance [30]. Insulin resistance can cause infertility and increased androgen levels, which exacerbate PCOS traits [31, 32]. In addition, the insulin-sensitizing drug metformin can improve menstrual disorder, promote ovulation, and elevate birth rate in PCOS, especially when accompanied by insulin resistance [33, 34]. Therefore, there should be a strong relationship between PCOS and insulin resistance. However, the underlying mechanism of insulin resistance in PCOS remains unclear. In the present study, we successfully established a model for PCOS with insulin resistance in rats by administrating letrozole and a high-fat diet to the rats. Letrozole is an aromatase inhibitor that blocks the conversion of androgen to estrogen. This results in elevated androgen levels, which is the main pathogenetic mechanism occurring in PCOS. Pubertal female rats were continuously treated with letrozole, inducing increased LH and T levels, abnormal estrous cycle, anovulation, increased ovarian weight, cysts, atretic follicles and no CL in adulthood [35, 36]. Furthermore, a high-fat diet in rats induces abnormal metabolism, including increased body weight, impaired glucose tolerance, and insulin resistance [37]. Thus, letrozole and a high-fat diet in rats contain both the reproductive and metabolic features associated with PCOS and insulin resistance.

Several miRNA, long-non-coding-RNA and circRNA expression profiling studies of PCOS have been conducted using different human components including serum, adipose tissue, uterine tissue, and follicular fluid [24, 38, 39]. Studies have implicated miRNAs in the pathogenesis of PCOS, including increased androgen [38, 39]. However, the exact role of miRNAs in the pathophysiology of PCOS with insulin resistance remains unclear.

The terms biological process, cellular component, and molecular function were predicted by a GO analysis of the differentially expressed miRNAs. Of these, the most significantly enriched term was metabolic process, indicating that these differentially expressed miRNAs participate in metabolism. Likewise, the most enriched pathway from the KEGG pathway analysis was also metabolism. It is known that insulin resistance, obesity, type 2 diabetes mellitus and dyslipidemia are common features of PCOS [1–3, 40]. Moreover, insulin resistance is an important characteristic of PCOS and contributes to the development of PCOS. Thus, these aberrantly expressed miRNAs may be the cause of insulin resistance in PCOS, though elucidating the underlying mechanism awaits further study.

We established miRNA expression profiles from the ovaries of PCOS rats with insulin resistance along with control rats. A total of 58 differentially expressed miRNAs in the PCOS ovaries exhibited significant changes compared with the control group, including 23 that were upregulated and 35 that were downregulated. Of note, we found that miR-3585-5p and miR-30-5p were significantly upregulated, and miR-146-5p was downregulated in the LEHF group, which was consistent with the deep sequencing results. MiR-30 was significantly upregulated in ovaries from PCOS with insulin resistance rats which is consistent with previous studies [28, 41, 42]. However, miR-146 was significantly upregulated in serum from PCOS patients compared with that of healthy women, which is inconsistent with our findings [28, 42]. This difference may be explained by the natural heterogeneity of PCOS as well as the different sequencing method employed. Interestingly, the expression of miR-146 was significantly decreased in peripheral blood mononuclear cells from type-2-diabetes patients compared with control subjects, and miR-146 was negatively correlated to insulin resistance [43]. This indicates that miR-146 may play a key role in the pathogenesis of insulin resistance in PCOS, and miR-146 may represent a novel diagnostic marker or therapeutic target for insulin resistance in PCOS. Therefore, miR-146 should be further investigated in this context.

However, this study has some limitations. In view of the methods and results we have got, this paper should be described as a pilot study, and this study is not large enough to do justice to the heterogeneity of PCOS. But these results might provide some assistance for further study of insulin resistance in PCOS. Therefore, we will make further experiments to study insulin resistance in PCOS and the comprehensive experiments will be obtained and reported in future.

In conclusion, we found 58 differentially expressed miRNAs in a rat model of PCOS with insulin resistance by deep sequencing analysis and verified the differential expression of miR-146-5p, miR-3585-5p and miR-30-5p by qRT-PCR. Furthermore, miR-146 may represent a novel diagnostic marker and therapeutic target for insulin resistance in PCOS. Additional studies are needed to identify the underlying mechanism of miRNA function in the development of PCOS with insulin resistance.
